# Oxford Nanopore Sequencing of Clinical DNA for Identification and Comparative Genomic Analysis of *Erysipelothrix piscisicarius*

**DOI:** 10.1155/tbed/5654145

**Published:** 2026-08-30

**Authors:** Rattagan Kajeekul, Thidathip Wongsurawat, Worarat Kruasuwan, Phichaya Sawankatat, Pattaraporn Nimsamer, Tantip Arigul, Athita Riwlord, Piroon Jenjaroenpun, Anusak Kerdsin

**Affiliations:** ^1^ Department of Medicine, Maharat Nakhon Ratchasima Hospital, Nakhon Ratchasima, Thailand, mnrh.go.th; ^2^ Medical Bioinformatics, Siriraj Genomics, Faculty of Medicine Siriraj Hospital, Mahidol University, Bangkok, Thailand, mahidol.ac.th; ^3^ Special Research Unit for Tropical Zoonoses and Antimicrobial Resistance Surveillance (TZARS), Kasetsart University, Bangkok, Thailand, ku.ac.th; ^4^ Clinical Microbiology Laboratory, Department of Medical Technology, Maharat Nakhon Ratchasima Hospital, Nakhon Ratchasima, Thailand, mnrh.go.th; ^5^ Faculty of Public Health, Kasetsart University, Chalermphrakiat Sakon Nakhon Province Campus, Sakon Nakhon, Thailand, ku.ac.th; ^6^ Faculty of Veterinary Science, Chulalongkorn University, Bangkok, Thailand, chula.ac.th; ^7^ Center of Excellence for Food and Water Risk Analysis (FAWRA), Department of Veterinary Public Health, Faculty of Veterinary Science, Chulalongkorn University, Bangkok, Thailand, chula.ac.th

**Keywords:** endocarditis, *Erysipelothrix piscisicarius*, genome, nanopore, septicemia

## Abstract

The genus *Erysipelothrix* comprises facultative anaerobic, nonspore‐forming, gram‐positive bacteria that can cause skin infections and severe diseases such as septicemia and endocarditis in humans. Although *E. rhusiopathiae* is the primary pathogen, other species may also be involved, necessitating accurate identification. However, 16S rDNA sequencing lacks sufficient resolution to differentiate among *Erysipelothrix* species. In this study, we used Oxford Nanopore Technology (ONT) to directly sequence low‐quality DNA extracted from heart valve tissue of a 66‐year‐old female patient with a fatal case of septicemia and aortic endocarditis. In contrast to 16S rDNA Illumina sequencing and matrix‐assisted laser desorption ionization time‐of‐flight mass spectrometry (MALDI‐TOF MS), which incorrectly identified the pathogen as *E. rhusiopathiae*, direct sequencing via ONT precisely identified *E. piscisicarius* as the cause of infection. About 1.47 Mb genome was retrieved from nanopore direct sequencing. Within the *E. piscisicarius* genome, we detected genes associated with virulence. Phylogenetic analysis showed that our strain clustered with a human‐derived *E. piscisicarius* strain from China and swine‐derived strains from Brazil. In conclusion, this study demonstrated that ONT can be used to sequence low‐quality DNA extracted directly from patient specimens, obtain a draft bacterial genome, and reliably distinguish between pathogenic species.

## 1. Introduction

*Erysipelothrix* is a genus of facultative anaerobic, nonsporulating, gram‐positive bacteria that includes the following species: *E. rhusiopathiae*, *E. tonsillarum*, *E. inopinata*, *E. larvae*, *E. piscisicarius*, *E. aquatica*, *E. anatis*, and *E. urinaevulpis* [1, 2]. Within this genus, *E. rhusiopathiae* is known to cause human diseases. Specifically, infection with *E. rhusiopathiae* often occurs after exposure of an open wound to contaminated water. This infection results in erysipeloid, an acute bacterial infection of the skin characterized by redness, swelling, low‐grade fever, blistering, and swollen lymph nodes [3, 4]. If left untreated, infection with *E. rhusiopathiae* can result in life‐threatening bacteremia and endocarditis.

Until recently, *E. rhusiopathiae* was believed to be the sole member of the *Erysipelothrix* genus responsible for human infection; however, in 2022, the first case of erysipeloid caused by *E. piscisicarius* was reported [5]. The patient was punctured in the finger when dealing with whiteleg shrimp (*Penaeus vannamei*) 3 days before the onset of the disease, and she was alive after treatment with piperacillin‐tazobactam [5]. To effectively treat infections caused by *Erysipelothrix* and for epidemiological tracking, we must rapidly and precisely identify the *Erysipelothrix* species responsible for infection. Culture‐based methods are often used to identify *Erysipelothrix* species [6] but are time‐consuming, complicated, and have low sensitivity. However, molecular identification or detection via PCR, real‐time PCR, sequencing, and metagenomic next‐generation sequencing has been used to resolve the problems associated with culturing methods [5–9].

In this study, we applied metagenomic next‐generation sequencing using the Oxford Nanopore Technology (ONT) platform to identify *E. piscisicarius* as the causative agent of the patient’s infection, despite the use of low‐quality DNA extracted from clinical specimens. Genomic analysis of *E. piscisicarius* revealed multiple genes associated with antimicrobial resistance and virulence, and comparative analyses were performed with other *E. piscisicarius* strains. Overall, our findings demonstrate that ONT can effectively sequence low‐quality DNA directly from patient samples, enabling the recovery of a draft bacterial genome suitable for downstream analyses.

## 2. Materials and Methods

### 2.1. Clinical Data Collection

Electronic medical data were used to examine the patient’s medical history. Age, sex, underlying diseases, initial clinical manifestations, laboratory data, treatment details, and clinical outcomes were recorded.

### 2.2. Microbiological Examination

Hemoculture was performed at the hospital laboratory. Bacteria from the hemoculture were subcultured onto sheep blood agar plates and incubated at 37°C for 24 h. If no bacterial growth was observed after 24 h, the plates were examined daily for up to 7 days. The following conventional biochemical tests were performed: gram staining, catalase production, hydrogen sulfide production on triple‐sugar iron agar, nitrate reduction, urease production, and motility. Carbohydrate utilization, including glucose, xylose, mannose, lactose, sucrose, and maltose, was also evaluated to support bacterial identification [10]. Matrix‐assisted laser desorption ionization time‐of‐flight mass spectrometry (MALDI‐TOF MS; MALDI Biotyper MSP Identification Standard Method 1.1) was used for complementary identification.

### 2.3. Genomic DNA Extraction and Metagenomic Next‐Generation Sequencing

Genomic DNA was initially extracted directly from the aortic valve tissue for diagnostic 16S rDNA metagenomic sequencing using the DNeasy Blood and Tissue Kit (Qiagen, Hilden, Germany), according to the manufacturer’s instructions. The extracted DNA was aliquoted for 16S rDNA metagenomic sequencing on the Illumina HiSeq 2500 platform at Ramathibodi Hospital Laboratory and for ONT metagenomic sequencing at the Siriraj Long‐Read Laboratory, Bangkok, Thailand. Host‐depletion procedures, such as saponin‐based selective lysis or differential lysis, were not performed before DNA extraction because the specimen had initially been processed for 16S rDNA sequencing, and only limited residual extracted DNA was available for ONT sequencing. Although host depletion would likely improve microbial read recovery in future metagenomic applications.

### 2.4. Oxford Nanopore Sequencing

DNA extracted from the aortic valve tissue was qualified using a Genomic DNA ScreenTape assay performed on a 4150 TapeStation according to the manufacturer’s instructions (Agilent Technologies, CA, USA). DNA was quantified with the Qubit 1× dsDNA High Sensitivity and Broad Range assay kit (Invitrogen, Thermo Fisher Scientific Inc., MA, USA) on a Qubit 4.0 Fluorometer (Invitrogen, Thermo Fisher Scientific Inc., MA, USA) according to the manufacturer protocols. Subsequently, we used 3 ng/μL of DNA and the Ligation Sequencing Kit (SQK‐LSK110, ONT, UK) to prepare a DNA library. The library was loaded into an R9.4.1 nanopore flow cell (FLO‐MIN106D) on a GridION device (ONT, UK) with the super accuracy mode for base calling. Real‐time data acquisition was performed using MinKNOW software (Version 23.04.6) with the minimum length parameter set to 20 to ensure that even small DNA fragments were sequenced. Real‐time base calling converted raw signals to DNA sequences using Guppy v6.5.7 using the SUP model.

### 2.5. Pathogen Identification Using Metagenomic Analysis

For real‐time data analysis, sequence reads were analyzed using the Antimicrobial Resistance v2023.04.26‐1808834 workflow via EPI2ME [11]. Briefly, reads were classified against a standard Centrifuge database [12]. Subsequently, minimap2 was used for antimicrobial resistant genes (AMRs) identification to align taxonomically classified reads against all reference sequences available in the Comprehensive Antibiotic Resistance Database (CARD) [13, 14]. Furthermore, FASTQ files were uploaded to the Chan Zuckerberg ID (CZ ID) cloud‐based platform (https://czid.org/) for pathogen identification using a nanopore metagenomics pipeline. Briefly, the uploaded data underwent a preprocessing step in which host reads were removed through a mapping process using Minimap2 [14] and assembled with metaFlye [15]. Finally, taxonomic classification was performed by aligning the contigs against the NCBI nucleotide (NT) and nonredundant protein (NR) databases.

### 2.6. De Novo Assembly Constrained by DNA Fragment Size and Yield

These sequences were used for pathogen identification. Subsequently, the reads were mapped to the human reference genome (T2T CHM13v2.0/hs1) to filter out the host DNA. The remaining nonhuman reads (Supporting Information 1: Table S1) were assembled de novo with Flye v2.9.2‐b1786 using the “‐‐meta” parameter, producing 21 contigs with a combined length of 191,052 bases and a GC content of 37.77% (Supporting Information 1: Table S1). To identify the assembled contigs, the largest contig was searched against the NT database using BLASTN. The best local‐alignment hit was the complete genome of the *E. piscisicarius* reference strain 15TAL0474 (CP034234.1), with 98.1% identity. To evaluate this identification beyond the local BLASTN hit to a genome‐level measure, we compared the de novo contigs against the 15TAL0474 genome using nucmer from the MUMmer package v3.23. This comparison gave an average NT identity (ANI) of 98.24% across the assembled contigs. Of the 191,052 assembled bases, 178,686 bases (93.53%) aligned to the 15TAL0474 genome, covering ~11.11% of the reference. This limited genome recovery may be explained by the short read‐length distribution as ~90% of the reads were shorter than 1000 bases and were filtered out during de novo assembly with Flye.

### 2.7. Reference‐Guided Consensus Assembly

Because de novo assembly discarded most of the short reads (<1000 bases) and recovered only a limited portion of the genome, we used a reference‐based approach that can reconstruct a draft genome from short, low‐coverage reads. We generated a polished consensus sequence against the *E. piscisicarius* reference strain 15TAL0474 (CP034234.1). Initial polishing was performed with Flye v2.9.2‐b1786 (“‐i 2 ‐‐polish‐target”), and final correction and gap filling were done with Medaka v2.1 (ONT) using the “‐r ‘N’” parameter to insert “N” placeholders. Genome completeness and contamination of the consensus were then assessed with CheckM2 v1.0.1.

The consensus draft genome was taxonomically classified using GTDB‐Tk v2.3.2. Genome annotation was performed using the NCBI Prokaryotic Genome Annotation Pipeline (PGAP v6.6) [16]. CARD (https://card.mcmaster.ca/analyze) was used to predict antimicrobial resistance‐associated genes, and virulence‐associated genes were analyzed using the Virulence Factor Database and MyDbFinder, including comparison with 16 virulence genes previously reported in *E. rhusiopathiae* [17, 18; http://www.mgc.ac.cn/cgi-bin/VFs/v5/main.cgi?func=VFanalyzer; https://cge.food.dtu.dk/services/MyDbFinder/]. Unless otherwise specified, default parameters were used.

A core‐genome SNP‐based phylogenetic tree was constructed using Snippy [19]. Finally, a phylogenetic tree was constructed from the core SNPs using IQ‐TREE based on maximum likelihood estimation with 1000 bootstrap replicates [20]. The tree was visualized using iTOL [21]. In addition, pangenome analyses of *E. piscisicarius* genomes, including our strain and isolates from China (CP034234) and Brazil (SBAR00000000, SCFT00000000), were performed using Panaroo (v1.6.0) [22], based on GFF3 annotations generated by Prokka (v1.15.6) [16]. To ensure high‐stringency gene clustering, the “strict” clean mode was employed with a 95% sequence identity threshold and a 70% protein family threshold. Gene clusters were categorized into core (present in 100% of isolates), shell (25%–75%), and cloud (unique to a single isolate) genomes. Genomic intersections among Thai, Chinese, and Brazilian isolates were identified via a gene presence‐absence matrix and visualized using UpSet R plots [23]. Unique genes were defined as those present exclusively in the target isolate (*n* = 1) and absent in all others (*n* = 0).

### 2.8. Accession Number

The genome sequence identified in this study has been deposited in the GenBank database (CP144372). The raw data have now been submitted under the SRA Accession SRR38297505.

## 3. Results

### 3.1. Case and Laboratory Examination

In 2024, a 66‐year‐old female with a medical history of autoimmune hemolytic anemia was diagnosed with essential hypertension. She was administered prednisolone (10–20 mg/day). Additionally, the patient had alcohol use disorder and presented with a 7‐day history of generalized erythematous papules and fever. Importantly, the patient, who had not recently traveled anywhere or encountered wildlife or ill humans, worked as a farmer and was often exposed to stagnant water. In addition, she swam in a rural canal 1 week before she came to the hospital.

**Figure 1:**
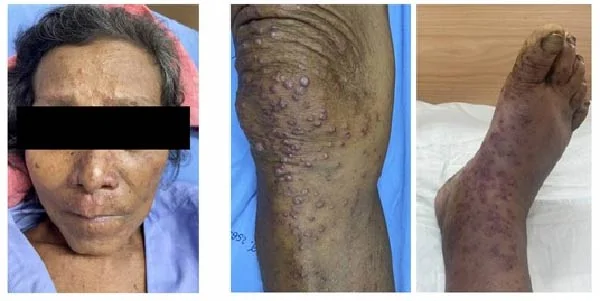
Characteristics of the patient infected with *Erysipelothrix piscisicarius* cutaneous eruption at presentation. Multiple erythematous to violaceous papules, some with pustules on her face, both knees, and both feet.

Upon physical examination, the woman had a 38°C fever and multiple red erythematous papules on the face and both legs, some of which contained pustules. She also had various purplish papules on her hands and feet (Figure 1). During the cardiovascular examination, a grade II short diastolic blowing murmur was detected in the aortic area, but neither heaves nor thrills were noted. Her chest examination was normal, and hepatosplenomegaly was not observed on the abdominal examination.

Because the patient presented with fever and rash, a disseminated infection with septic vasculitis, leukaemic cutis, and leucocytoclastic vasculitis was suspected. Additionally, infective endocarditis was a concern because she had an audible murmur. Chest radiography showed mild cardiomegaly but no evidence of parenchymal abnormalities. No evidence of cardiomegaly or abnormal parenchyma was observed, and an electrocardiogram revealed sinus tachycardia, a normal axis, the absence of hypertrophy, and nonspecific ST‐ and T‐wave abnormalities.

Initial laboratory studies indicated that the patient had an elevated white blood cell count of 13,900 cells/mm^3^ (reference range: 4500–11,000 cells/mm^3^), which predominantly consisted of neutrophils (86.1%), and a decreased platelet count of 138,000 cells/µL (reference range: 150,000–400,000/µL). The patient’s blood urea nitrogen level was 8.2 mg/dL (reference range: 8–20 mg/dL), and her serum creatinine level was 0.64 mg/dL (reference range: 0.8–1.3 mg/dL). Other serum values were as follows: 168 U/L aspartate aminotransferase (reference range: 10–40 U/L), 68 U/L alanine transaminase (reference range: 7–56 U/L), 123 U/L alkaline phosphatase (reference range: 44–147 U/L), 1.1 mg/dL total bilirubin (reference range 0.1–1.2 mg/dL), 0.3 mg/dL direct bilirubin (reference range: 0.0–0.3 mg/dL), 2.8 g/dL and albumin (reference range: 3.4–5.4 g/dL). The patient received empirical therapy with intravenous ceftazidime and clindamycin.

After a positive hamoculture, a microbiological examination was performed. Pinpoint colonies on 5% sheep blood and chocolate agar plates showed alpha‐hemolysis, and gram‐positive bacilli were observed. Conventional biochemical tests were used to identify the *Erysipelothrix* species. Using MALDI‐TOF MS, the *Erysipelothrix* species was determined to be *E. rhusiopathiae*. Unfortunately, the isolated bacterium was not stored after the report; therefore, no isolates were available for further study.

The antimicrobial therapy was switched to intravenous penicillin G. An echocardiogram was conducted after *E. rhusiopathiae* was identified. The echocardiogram revealed mild dilatation of the left atrium and left ventricle (LV), eccentric hypertrophy of the LV, preserved systolic function of the LV (EF 71.4% by plane), normal size of the right atrium and ventricle, a trileaflet aortic valve measuring 0.84 cm × 1.44 cm with severe aortic regurgitation, and the absence of pericardial effusion.

On the ninth day of hospitalization, surgery was performed to replace the aortic valve. Surgical examination revealed a normally sized ascending aorta and a normal‐looking valve, except for the presence of a 1 cm × 1.5 cm vegetation at the noncoronary cusp and a 1 cm × 1.2 cm vegetation at the left coronary cusp, which resulted in aortic regurgitation. During this period, tissue from the aortic valve was extracted and sent for molecular identification (metagenomic next‐generation sequencing). 16S rDNA metagenomic sequencing using the Illumina sequencing platform identified *E. rhusiopathiae* with 99% coverage and 100% identity based on BLASTN analysis; however, ONT metagenomic sequencing revealed *E. piscisicarius* with a high score and all read matching.

Following the surgical procedure, the patient continued taking antibiotics, and the bacteria failed to grow in hemoculture, which was consistent with the patient’s fever. However, 10 days after the surgical procedure, the patient developed fever due to hospital‐acquired pneumonia caused by *Acinetobacter baumannii*. Consequently, colistin was administered; however, her condition deteriorated, and she died.

### 3.2. Human *E. piscisicarius* Cases

To date, only two human cases have been reported: the first, an erysipeloid case described in China [5], and the second, a case of septicemia with infective endocarditis presented here. As summarized in Table 1, comparison of risk factors, clinical manifestations, investigations, and treatment highlights the broad clinical spectrum and emerging zoonotic potential of *E. piscisicarius* infection.

**Table 1:** Comparison of clinical manifestations and epidemiological characteristics of human *Erysipelothrix piscisicarius* infections.

Category	This case (Thailand, 2024)	Huang et al. [5] (China, 2022)
Country	Thailand	China
Patient Age/sex	66‐year‐old female	72‐year‐old female
Underlying conditions	Autoimmune hemolytic anemia, hypertension	Not clearly stated
Exposure history	Farmer Swam in rural canal 1 week before illness	Finger puncture while washing shrimp 3 days before illness
Suspected transmission route	Environmental water exposure	Traumatic inoculation from shrimp handling
Zoonotic link	Aquatic environment	Shrimp (aquaculture product)
Immunocompromised status	Yes (chronic steroids used)	Not reported
Initial presentation	Fever and generalized erythematous papules	Fever with chills of unknown origin
Systemic symptoms	Fever, murmur suggestive of endocarditis	Fever, headache, vertigo, drowsiness, hypotension, tachypnea
Cutaneous manifestations	Multiple erythematous to violaceous papules with pustules (face, legs, hands, feet)	Localized red swollen mass on left thumb (1 cm × 0.5 cm)
Cardiac involvement	Yes—severe aortic regurgitation with valve vegetation	No cardiac involvement
Severity	Severe invasive disease	Localized skin lesion
Microbiological identification by MALDI‐TOF	*E. rhusiopathiae*	*E. rhusiopathiae*
Molecular identification	*E. piscisicarius* by ONT metagenomics	*E. piscisicarius* by mNGS and WGS
Antimicrobial susceptibility	Not performed	Susceptible to piperacillin‐tazobactam, imipenem, ciprofloxacin
Treatment	Ceftazidime plus clindamycin switch to penicillin G	Piperacillin‐tazobactam
Outcome	Death form hospital‐acquired pneumonia due to *Acinetobacter baumannii*	Recovered and discharged

In the initial case reported by Huang et al. [5] (China, 2022), infection followed a traumatic puncture sustained while handling shrimp (*Penaeus vannamei*), resulting in localized erysipeloid with systemic inflammatory features and a favorable outcome after antimicrobial therapy. In contrast, the present case from Thailand (2024) involved a 66‐year‐old immunocompromised woman with exposure to stagnant freshwater, who developed invasive septicemia complicated by aortic valve endocarditis and ultimately died from secondary nosocomial pneumonia.

These cases suggest that aquatic exposure, whether through handling marine organisms or contact with natural water sources, is a key epidemiological risk factor. Notably, both infections were initially misidentified as *E. rhusiopathiae* using conventional methods and were only accurately identified through metagenomic or whole‐genome sequencing, underscoring the limitations of MALDI‐TOF MS in differentiating closely related *Erysipelothrix* species.

Collectively, these findings indicate that *E. piscisicarius* can cause both localized cutaneous disease and severe invasive infection, particularly in vulnerable hosts, and emphasize the need for increased clinical awareness and improved species‐level diagnostic approaches.

### 3.3. Nanopore Sequencing of Degraded and Low‐Quality DNA

**Figure 2:**
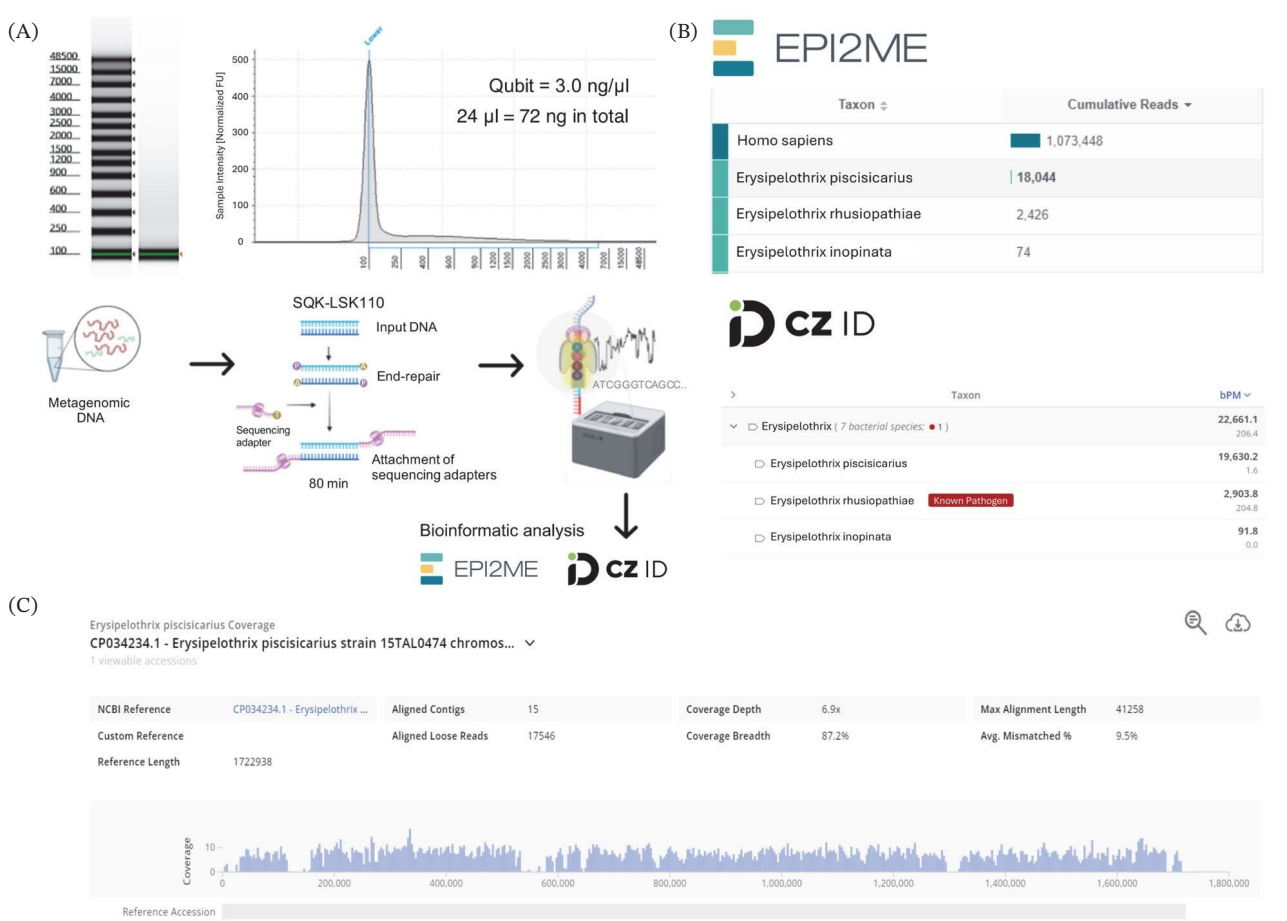
Process of ONT and bioinformatics analysis of *Erysipelothrix piscisicarius*. (A) Quality check of *Erysipelothrix piscisicarius* DNA extracted and process of metagenome analysis. (B) Real‐time taxonomic identification by EPI2ME and CZ ID. (C) Contigs and annotations from the CZ ID pipeline, shown as evidence for the identification.

Figure 2 illustrates the nanopore sequencing process. DNA quality, which encompasses the concentration, purity, and size, is crucial for the success of nanopore sequencing. In our study, the DNA input quality did not align with the standard nanopore recommendation of 100 ng of high molecular weight (HMW) DNA. The sample contained 72 ng of DNA, with most fragments under 1000 bases, which is far below the HMW DNA quality standards. Nevertheless, we sequenced our DNA input with a new short‐fragment sequencing feature using ONT, which enabled the sequencing of DNA fragments as short as 20 bases and allocated an entire flow cell to a single sample (Figure 2). Our results indicated that the DNA size distribution was ~300–400 base pairs. Low‐quality DNA affected the sequencing yield in nanopore sequencing but did not affect the quality of the sequenced reads. This was used for downstream data analysis. Details of the nanopore sequencing results are summarized in Supporting Information 1: Table S1.

### 3.4. Nanopore‐Based Metagenomic Analysis

Nanopore sequencing generated ~1,096,000 reads, which were used for the real‐time analysis of EPI2ME and CZ ID (Figure 2). Of all the reads, most of the filtered reads belonged to humans (*Homo sapiens*); however, both EPI2ME and CZ ID identified *E. piscisicarius* (~1.6%), *E. rhusiopathiae* (~0.2%), and *E. inopinata* (~0.01%) as the top three similar species. Moreover, the consensus genome assembly of *E. piscisicarius* via CZ ID identified 15 contigs, with ~7× genome coverage and 87.2% coverage breadth, whereas only three contigs were constructed for the known pathogen *E. rhusiopathiae* (Figure 2).

### 3.5. Genome Analysis

The reference‐guided consensus produced a 1.47 Mb draft genome with a GC content of 37.42%. Positions without read support were masked with “N,” so the consensus carried only bases supported by the sequencing data. CheckM2 estimated the consensus to be 77.76% complete with 4.18% contamination, consistent with a medium‐quality draft genome. We then used GTDB‐Tk to assess the consensus against the closest reference genome (GCF_003931795.1, *E. piscisicarius* strain 15TAL0474), which reports both ANI and the genome coverage of the consensus relative to the closest species. The consensus showed a 98.31% ANI and a 0.98 alignment fraction. This ANI was consistent with the 98.24% ANI obtained independently from the reference‐free de novo assembly using MUMmer against 15TAL0474. In addition, the reference‐guided consensus showed 99.78% ANI to the de novo assembly, supporting the consensus retained patient‐derived sequence information rather than simply reproducing the reference genome. Together, the read‐level metagenomic classification, de novo assembly comparison, and concordant consensus‐based classification supported the identification of the pathogen as *E. piscisicarius*. CARD screening did not identify high‐confidence acquired resistance genes. The only matches were low‐identity, partial homologs of van‐cluster accessory genes (strict hits, 36%–57% identity) without the resistance ligase or a complete operon, consistent with chromosomal homologs rather than acquired vancomycin resistance. Because no isolate was preserved for susceptibility testing, these findings were not confirmed phenotypically.

**Figure 3:**
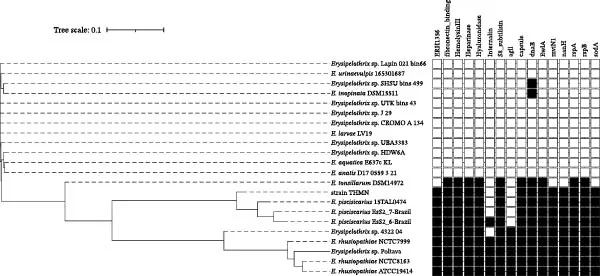
Core‐genome SNP phylogenetic tree of representative *Erysipelothrix* spp., including strain THMN in this study. Black boxes indicate the presence of virulence genes, whereas white boxes indicate their absence.

Putative virulence‐associated genes in the *E. piscisicarius* genome were predicted using the Virulence Factor Database and compared with the virulence genes previously reported in *E. rhusiopathiae*. For strain THMN, patient‐derived reads were mapped back to the consensus genome to confirm support for the reported virulence genes. The median read depth across these genes ranged from 5x to 13x (Supporting Information 2: Table S2). The putative virulence‐associated genes included those for adherence, enzymes, toxins, capsular polysaccharides, secretion systems, and surface proteins (Figure 3). *Erysipelothrix rhusiopathiae* has 16 known virulence genes, including *algI*, *cap* locus, *dnaB*, ERH‐1356, *ewlA*, *fbpA*, *hep*, *hlyA*, *hlyIII*, *intl-like*, *mviN1*, *nanH*, *rspA*, *rspB*, *sodA*, and *sub* [17], 14 of the 16 virulence genes were identified in the genomes of *E. piscisicarius* strains THMN, 15TAL0474, and EsS2_7‐Brazil (Figure 3). Only two virulence genes, *intl* (internalin) and *algI* (alginate O‐acetyltransferase), were not detected in these genomes. In contrast, *E. piscisicarius* strain EsS2_6‐Brazil lacked only the *algI* gene. Overall, our data suggest that *E. piscisicarius* is likely to exhibit a level of virulence comparable to that of *E. rhusiopathiae*.

Core‐genome SNP–based phylogenetic analysis, including our strain THMN and *Erysipelothrix* spp., demonstrated that THMN is closely related to *E. piscisicarius* strain 15TAL0474 (human, China) and clusters with swine *E. piscisicarius* (formerly *Erysipelothrix* sp. strain 2) and strains EsS2‐6 and EsS2‐7 from Brazil (Figure 3). These findings confirm that strain THMN belongs to *E. piscisicarius*. Although the genomic similarity among different *Erysipelothrix* species is relatively low, the inclusion of representative species allows the phylogenetic position of *E. piscisicarius* to be evaluated within the broader genus and demonstrates its distinct clustering (Figure 3).

**Figure 4:**
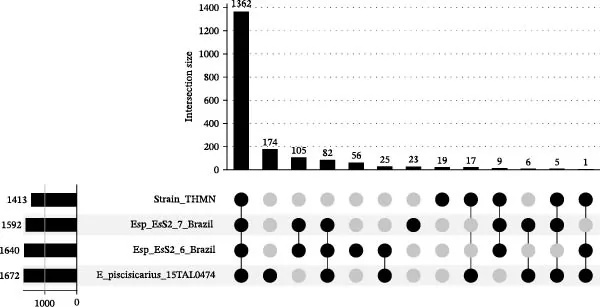
UpsetR showing the number of genes that are shared and unique among the genomes of four *E. piscisicarius* strains.

A comparative genomic analysis of strain THMN with strains 15TAL0474, EsS2_6‐Brazil, and EsS2_7‐Brazil identified 1362 coding sequences shared among all four genomes (Figure 4, Supporting Information 3: Table S3). This high number of shared coding sequences supports broad gene‐level coverage of the THMN draft genome generated from the patient‐derived ONT reads. However, because THMN was reconstructed using a reference‐guided approach with strain 15TAL0474 as the reference, strain‐specific genes, hypothetical proteins, and unique coding sequences should be interpreted with caution. Although the analysis identified 19 THMN‐specific coding sequences, most encoded hypothetical proteins. Therefore, these findings should be regarded as putative because of the low‐coverage, reference‐guided nature of the assembly. Swine strains EsS2_6 (Brazil) and EsS2_7 (Brazil) harbored 56 and 23 unique coding sequences, respectively, encoding hypothetical proteins, transport proteins, regulatory proteins, metabolic enzymes, and mobile genetic elements (Figure 4, Supporting Information 3: Table S3). In contrast, the human strain 15TAL0474 harbored 174 unique coding sequences encoding enzymes involved in metabolism, DNA replication and repair, signal transduction–related regulatory proteins, insertion sequence transposases, and hypothetical proteins (Figure 4, Supporting Information 3: Table S3). The identified unique genes should not be interpreted as definitive lineage‐ or host‐specific determinants. Instead, they represent candidate accessory genes that may reflect recent genome diversification driven by horizontal gene transfer, mobile genetic elements, or other genome remodeling events.

## 4. Discussion

We report a fatal case of endocarditis and septicemia caused by *E. piscisicarius*. To the best of our knowledge, this is the second case in which a human was infected with *E. piscisicarius*, which was likely caused by exposure to stagnant water. Given our findings, emerging pathogens such as *E. piscisicarius* should be considered along with known pathogens such as *E. rhusiopathiae* when patients have a history of exposure to natural water or aquatic animals.

Although 16S rDNA is commonly used as a marker to detect unknown bacteria in microbial communities, a recent comparative genome study of *Erysipelothrix* suggested that full‐length 16S rDNA cannot be used to distinguish between *Erysipelothrix* species [1]. In this study, 16S rDNA sequencing, initially conducted using the Illumina technology, detected an unidentified bacterial species in a clinical specimen and identified the genus *Erysipelothrix*. Given the genus, the infectious agent was assumed to be *E. rhusiopathiae*, which is commonly associated with superficial skin infections that escalate to bacteremia and endocarditis in humans [24, 25]. Consistent with the Illumina sequencing results, the isolate was identified via MALDI‐TOF MS as *E. rhusiopathiae*. This may be due to the MALDI‐TOF MS database, which probably insufficiently covers rare species.

Unfortunately, the isolate recovered from blood culture in our study was not stored; therefore, confirmation of the genus and species by whole‐genome sequencing of the cultured isolate was not possible. Nevertheless, we performed nanopore‐based metagenomic sequencing using DNA extracted directly from the clinical specimen. This approach yielded a draft genome of *E. piscisicarius*, demonstrating the feasibility of recovering bacterial genomic information directly from clinical material. In this case, most sequencing reads were of human origin, which limited the depth of coverage available for the pathogen genome. In future direct clinical sequencing applications, host‐depletion procedures performed before DNA extraction could reduce the human DNA background and improve pathogen genome recovery when sufficient specimen material is available. Despite these limitations, the draft genome enabled further analysis of the identified pathogen.

As mentioned previously, ONT metagenomic sequencing and Illumina/MALDI‐TOF MS yielded discordant results, and similar inconsistencies have been reported [5]. Considering these inconsistencies, 16S rDNA sequencing and MALDI‐TOF MS to identify *E. piscisicarius* should be used with caution. A previous study demonstrated the limitations of 16S rDNA in identifying the *Erysipelotrix* genus [26]. However, the MALDI‐TOF MS library did not include *E. piscisicarius*. With the draft genome obtained by ONT, identification at the species level is more accurate than the identification of one gene alone, such as 16S rDNA. A polyphasic approach or higher‐resolution techniques such as whole‐genome sequencing or metagenomic sequencing should be used for correct identification. However, accurate species identification does not change the clinical management of *Erysipelothrix* infections because *Erysipelothrix* spp. remains highly susceptible to β‐lactam antibiotics. Consequently, β‐lactams continue to be the recommended treatment [3, 27].

This study has limitations. The de novo assembly recovered only 191,052 bp, ~13% of the genome, so the draft genome relied largely on the reference‐guided consensus. The consensus was a medium‐quality draft, with 77.76% completeness and 4.18% contamination, according to CheckM2. Positions without read support, representing 0.28% of the consensus genome, were coded as “N” rather than being inferred directly from the reference genome. Although the consensus agreed with the reference‐free de novo assembly at 99.78% ANI, genome‐wide interpretations depend in part on the reference‐guided assembly strategy. Given the limited number of publicly available *E. piscisicarius* genomes, the observed accessory genome variation may be influenced by undersampling. Additional genome sequences from diverse hosts and geographical regions are required to determine whether these differences represent lineage‐specific characteristics or simply reflect incomplete sampling of the species diversity.

The pangenome analysis complements the phylogenetic findings by revealing differences in accessory genome composition among closely related strains. However, because THMN was reconstructed using a reference‐guided approach with strain 15TAL0474 as the reference, the detection and interpretation of strain‐specific or unique coding sequences should be considered limited. The identified unique genes should be interpreted cautiously as accessory gene content can be influenced by genome assembly, annotation, and pangenome inference. Rather than representing definitive lineage‐ or host‐specific determinants, these genes should be considered candidate accessory genes that may reflect recent genomic diversification and genome plasticity [28, 29]. Notably, the majority encoded hypothetical proteins or mobile genetic element‐associated functions, including insertion sequence transposases and recombinases, suggesting that recent genome remodeling through horizontal gene transfer, gene gain and loss, and recombination has played a greater role than the acquisition of established virulence or host‐adaptive determinants [28, 29]. Together with phylogenetic analysis, these findings indicate that closely related strains continue to diversify through dynamic changes in their accessory genomes during adaptation to different host environments.

Nanopore sequencing also allowed genome‐based screening for antimicrobial resistance genes. We did not find high‐confidence acquired resistance determinants in the *E. piscisicarius* genome. The only CARD hits were low‐identity partial homologs of van‐cluster accessory genes, without the resistance ligase or a complete operon, which are most consistent with chromosomal homologs. Acquired resistance has, however, been reported in other *Erysipelothrix* strains. For example, analysis of *E. rhusiopathiae* strain 319078 identified genes predicted to encode class C and metallo‐beta‐lactamases [30], and a separate study reported a multidrug‐resistant *E. rhusiopathiae* carrying multiple resistance genes [31]. Phenotypic testing would be needed to assess the resistance in any individual genome.

Currently, *Erysipelothrix* sp. strain 2, including strains EsS2‐6 and EsS2‐7, has been officially designated as *E*. *piscisicarius* [32, 33]. Our analysis demonstrated that strain THMN, together with strains 15TAL0474, EsS2‐6, and EsS2‐7, clustered within the same phylogenetic group. These strains were isolated from humans and pigs, indicating that *E. piscisicarius* has expanded its host range beyond fish and is emerging as a pathogen capable of causing disease in terrestrial animals and humans [5, 32, 33].

The *E. piscisicarius* genome identified in this study contains putative virulence genes that participate in the biosynthesis of capsular polysaccharides, secretion systems, adhesive surface proteins, and enzymes or toxins involved in pathogenesis, such as cytolysin, heparinase, hyaluronidase, hemolysin‐III, and neuraminidase. Also, studies suggest that the *E. rhusiopathiae* capsule, along with *E. rhusiopathiae* enzymes and surface proteins, contribute to its virulence [34, 35]. For example, neuraminidase plays a significant role in the attachment and subsequent invasion of host cells [36]. *E. rhusiopathiae* adhesive surface proteins such as RspA and RspB can bind to polystyrene, fibronectin, and Type I and IV collagen [37].

Two additional studies found that only 13% of tiger barbs challenged with *E. piscisicarius* survived [1], and the pathogenesis of piscine erysipelosis in tiger barbs has been elucidated [38]. A recent experimental study demonstrated that challenge with *E. piscisicarius* in pigs results in mild clinical signs, including transient fever and characteristic rhomboid skin lesions, similar to classical swine erysipelas [32]. Collectively, *E. piscisicarius* is pathogenic and virulent; however, specific mechanisms driving its virulence remain poorly understood. Current research highlights several critical gaps that require further investigation. The emergence of *E. piscisicarius* underscores the urgent need for a more sophisticated approach of accurate species‐level diagnostics, active surveillance, and evaluation of current vaccine coverage strategies to manage *Erysipelothrix* infections across both aquatic and terrestrial industries.

## 5. Conclusion

Our study revealed that septicemia and aortic endocarditis cases in a female patient could be attributed to *E. piscisicarius*. We also demonstrated the usefulness of the nanopore technique for direct sequencing of low‐quality DNA extracted directly from a patient specimen, which is beneficial to clinical practice, such as treatment procedures. Our findings demonstrated that this nanopore sequencing approach can be used to obtain a draft bacterial genome directly from low‐quality extracted DNA. This advanced methodology facilitates cost‐effective sequencing of individual samples, eliminating the need to aggregate multiple samples for batch processing, as required by Illumina sequencing techniques. In addition, genomic analysis revealed that *E. piscisicarius* is potentially as virulent as *E. rhusiopathiae* as it harbors numerous virulence factors also identified in *E. rhusiopathiae*.

## Author Contributions

Rattagan Kajeekul, Anusak Kerdsin, and Thidathip Wongsurawat conceptualized the study design, methodology, writing – original draft, writing – review and editing. Thidathip Wongsurawat, Piroon Jenjaroenpun, Worarat Kruasuwan, Phichaya Sawankatat, Pattaraporn Nimsamer, and Tantip Arigul performed nanopore sequencing and analysis. Athita Riwlord performed microbiological analysis. Anusak Kerdsin, Thidathip Wongsurawat, Piroon Jenjaroenpun, Phichaya Sawankatat, and Rattagan Kajeekul analyzed and interpreted the data.

## Funding

This work was supported by a grant from the Kasetsart University Research and Development Institute (KURDI) (Grant FF(KU‐SRIU)16.67).

## Disclosure

All authors read and approved the final manuscript. Following the use of ChatGPT, the authors carefully reviewed, revised, and edited the manuscript as necessary. The authors accept full responsibility for the accuracy, integrity, and content of the final published work. In addition, the manuscript was edited by a native English speaker at KURDI.

## Ethics Statement

Ethical approval for this study was obtained from the Ethics Committee of Maharat Nakhon Ratchasima Hospital in Thailand. When evaluating the medical records, the authors followed a protocol approved by the Ethics Committee. This study was conducted in accordance with the Principles of the Declaration of Helsinki. The approval number is 028/2024. Written informed consent was obtained from the patient’s relatives.

## Conflicts of Interest

The authors declare no conflicts of interest.

## Supporting Information

Additional supporting information can be found online in the Supporting Information section.

## Supporting information


**Supporting Information 1** Table S1: Summary of sequencing data, including the number of reads after host genome removal, de novo assembly statistics, and the size and sequencing depth of each assembled contig.

**Supporting Information 2** Table S2: Sequencing metrics for the *E. piscisicarius* strains THMN, 15TAL0474, EsS2‐6, and EsS2‐7.

**Supporting Information 3** Table S3: Unique genes identified in *E. piscisicarius* strain THMN, EsS2‐6, EsS2‐7, and 15TAL0474.

## Data Availability

The data that support the findings of this study are available from the corresponding author upon reasonable request.
